# Applying High-Resolution Impedance Manometry for Detecting Swallowing Change in Anterior Cervical Spine Surgery Patients

**DOI:** 10.3389/fsurg.2022.851126

**Published:** 2022-03-16

**Authors:** Chih-Jun Lai, Ya-Jung Cheng, Dar-Ming Lai, Chun-Yu Wu, Wen-Ting Chang, Fon-Yih Tsuang

**Affiliations:** ^1^Institute of Epidemiology and Preventive Medicine, National Taiwan University, Taipei, Taiwan; ^2^Department of Anesthesiology, National Taiwan University Hospital, Taipei, Taiwan; ^3^Department of Anesthesiology, College of Medicine, National Taiwan University, Taipei, Taiwan; ^4^Department of Anesthesiology, National Taiwan University Cancer Center, Taipei, Taiwan; ^5^Division of Neurosurgery, Department of Surgery, National Taiwan University Hospital, Taipei, Taiwan

**Keywords:** anterior cervical spine surgery, high-resolution impedance manometry, hypopharynx, perioperative swallowing physiology, upper esophageal sphincter

## Abstract

**Background:**

Objectively detecting perioperative swallowing changes is essential for differentiating the reporting of subjective trouble sensations in patients undergoing anterior cervical spine surgery (ACSS). Swallowing indicates the transmission of fluid boluses from the pharynx (velopharynx, oropharynx, and hypopharynx) through the upper esophageal sphincter (UES). Abnormal swallowing can reveal fluid accumulation at the pharynx, which increased the aspiration risk. However, objective evidence is limited. High-resolution impedance manometry (HRIM) was applied for an objective swallowing evaluation for a more detailed analysis. We aimed to elucidate whether HRIM can be used to detect perioperative swallowing changes in patients undergoing ACSS.

**Methods:**

Fourteen patients undergoing elective ACSS underwent HRIM with the Dysphagia Short Questionnaire (DSQ, score: 0–18) preoperatively (PreOP), on postoperative at day 1 (POD1), and postoperative at day seven (POD7). We calculated hypopharyngeal and UES variables, including hypopharyngeal mean peak pressure (PeakP) and UES peak pressure, representing their contractility (normal range of PeakP, 69–280 mmHg; peak pressure, 149–548 mmHg). The velopharynx-to-tongue base contractile (VTI) was also calculated (normal range, 300–700 mmHg.s.cm), indicating contractility. The swallowing risk index (SRI) from HRIM combined with four hypopharyngeal parameters, including PeakP, represents the global swallowing function (normal range, 0–11). A higher SRI value indicated higher aspiration.

**Results:**

SRI was significantly higher on POD1 (10.88 ± 5.69) than PreOP (6.06 ± 3.71) and POD7 (8.99 ± 4.64). In all patients, PeakP was significantly lower on POD1 (61.8 ± 18.0 mmHg) than PreOP (84.9 ±34.7 mmHg) and on POD7 (75.3 ± 23.4 mmHg). The UES peak pressure was significantly lower on POD1 (80.4 ± 30.0 mmHg) than PreOP (112.9 ± 49.3 mmHg) and on POD7 (105.6 ± 59.1 mmHg). Other variables, including VTI, did not change significantly among the three time points. DSQ scores were 1.36, 3.43, and 2.36 at PreOP, POD1, and POD7 respectively.

**Conclusions:**

With similar trends in DSQ and SRI, swallowing was significantly decreased on POD1 because of decreased hypopharyngeal and UES contractility but recovered to the preoperative state on POD7 after ACSS. Applying HRIM is superior to DSQ in detecting mechanisms and monitoring the recovery from swallowing dysfunction.

**Clinical Trial Registration:**

The study was registered at ClinicalTrials.gov (NCT03891940).

## Introduction

**Figure 1:**
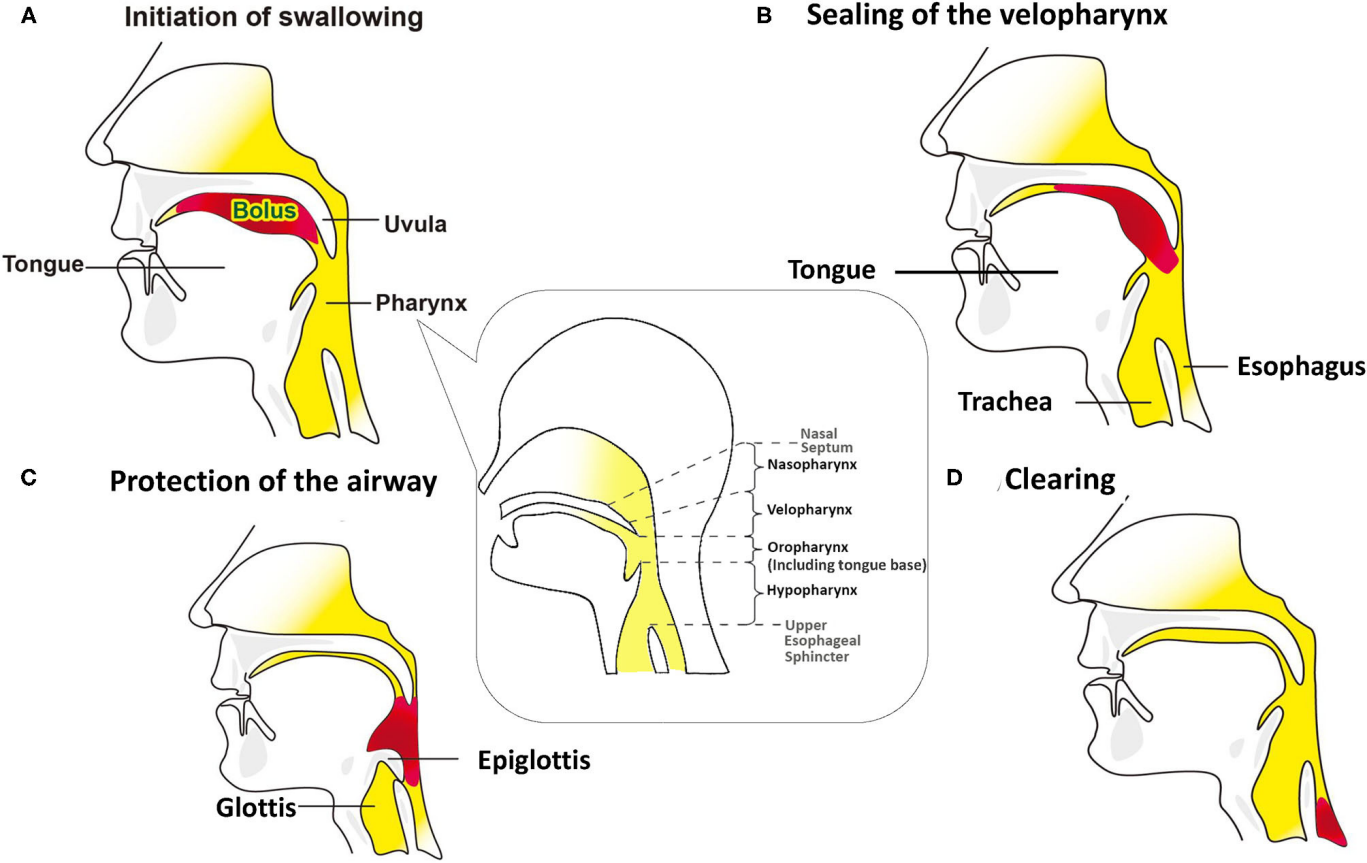
Swallowing mechanisms of bolus through the oral cavity into the esophagus. The red pattern in **(B–D)** depicts the bolus in the same way that does in the **(A)**. **(A)** The bolus is kept in the oral cavity, initiating the swallowing process. The figure in the dialog box below the pharynx illustrates that the pharynx includes the velopharynx, oropharynx and hypopharynx. The base of tongue is part of the oropharynx, and usually not visible when the mouth is open. **(B)** During the pharyngeal phase, the velopharynx, oral cavity, and larynx are sealing, and the bolus is transmitted through the pharynx and into the esophagus by pharyngeal peristalsis. **(C)** Airway protection is essential during the swallowing process, which includes tilting back of epiglottis, closing the laryngeal vestibule, trucking of the sealed airway under the tongue base from the bolus path, and the neuronal suppression of respiration while the bolus passes through the pharynx. **(D)** As the bolus passing into the esophagus, the airway reopens.

Objective detection of perioperative changes in swallowing is essential for differentiating only subjectively trouble swallowing sensations in patients undergoing anterior cervical spine surgery (ACSS). Normal effective swallowing is defined as the ability of pharyngeal peristalsis to transfer fluid boluses through the velopharynx, oropharynx, hypopharynx, and through the upper esophageal sphincter (UES) into the esophagus (Figure 1) (1). If one of these mechanisms is dysfunctional, it will cause bolus accumulation in these regions and increase the aspiration risk (2, 3).

Anesthesia and surgical manipulation of the ACSS may interfere with postoperative swallowing by affecting both sensory and motor functions. The trouble with swallowing is a subjective sensation and is the most common compliant in patients undergoing ACSS (4–8). However, objective evidence from previous studies assessing perioperative changes in swallowing is limited. Most previous investigators relied on patient self-report questionnaires, which are subjective and do not detect mechanisms of swallowing dysfunction (6). Some objective evaluation tools still have many shortcomings, such as radiation exposure in videofluoroscopy and inability to quantify swallowing physiology in the fiberoptic endoscopic evaluation of swallowing (9). In addition, the most concerning aspect of these tools is the inability to detect and differentiate the dysfunction of swallowing mechanisms. Early detection and differentiation of perioperative abnormal swallowing problems could help early treatment precisely (10).

High-resolution impedance manometry (HRIM) is a novel and reproducible tool for objectively assessing swallowing function using multiple pressure sensors and impedance channels (9, 11–14). In addition to multiple channels to measure pressure progression for effective pharyngeal peristalsis, a fluid bolus passing through the pharyngeal region can be detected through a low-impedance signal (12, 15). It could assess whether the muscle groups at the velopharynx, oropharynx, hypopharynx and UES could contract and open effectively and smoothly without resistance. HRIM also extends many parameters to measure them. In this study, we aimed to elucidate whether HRIM can be used to detect perioperative swallowing changes in patients undergoing ACSS. The data obtained by HRIM and subjective questionnaires on postoperative day one (POD1) and postoperative day seven (POD7) were compared with those collected preoperatively (PreOP). Swallowing dysfunction was determined by differentiating the affected physiology and recovery time.

## Participants and Methods

### Participants

Written informed consent was obtained from all patients participating in this study. The study was approved by the Ethics Committee of the National Taiwan University Hospital (No. 201901089RINC) and was registered at ClinicalTrials.gov (NCT03891940). Consecutive patients who underwent ACSS were eligible for enrollment. The study population included patients aged 20–80 years who underwent surgery for degenerative or traumatic conditions involving any cervical level. Patients were excluded from the study if they (1) had any major systemic disease, such as congestive heart failure, liver cirrhosis, end-stage renal disease, or malignancy; (2) were at risk of difficult ventilation or intubation; (3) were pregnant; (4) exhibited coagulopathy.

### Equipment: High-Resolution Impedance Manometry

**Figure 2:**
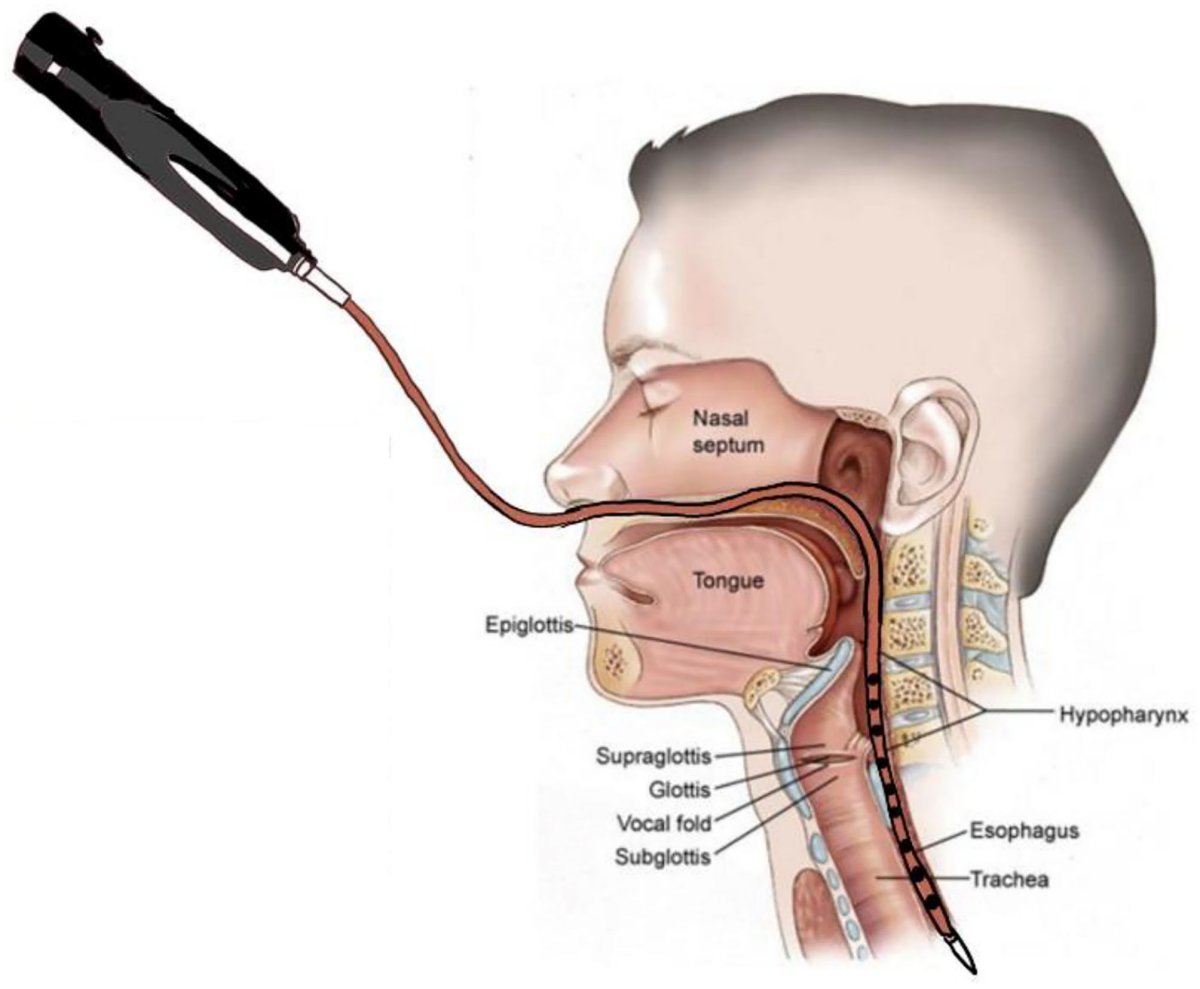
Swallow function recorded by High-Resolution Impedance Manometry (HRIM). The HRIM tube is shown in a patient's pharyngeal segment.

Manometric studies were completed using a 10 Fr outer diameter solid-state assembly with 36 circumferential pressure sensors at 1 cm intervals and 12 impedance segments at 2 cm intervals (MMS, Enschede, the Netherlands). Before each recording, the catheter was calibrated to the atmospheric pressure, according to the manufacturer's instructions. After a minimum 8 h of fasting, patients were intubated, and the catheter was positioned with sensors straddling the entire pharyngoesophageal segment (Figure 2). Pressure and impedance data were acquired at 20 Hz (Solar GI acquisition system, MMS, The Netherlands) with the patient sitting upright.

### Surgical Technique

Patients were intubated with general anesthesia and remained supine on the operation table with the neck extended. The patient received 1 g of cefazolin as a prophylactic antibiotic within 1 h of the incision. A skin surface landmark was used to determine the incision site for the affected cervical segment. The operating surgeon was right-handed; and thus, the surgery was performed on the right side of the patient. A horizontal linear skin incision of ~4 cm was made in the skin and increased from the midline to the anterior border of the right sternocleidomastoid muscle. After careful dissection of the subcutaneous soft tissue, the anterior border of platysma muscle was exposed. The platysma muscle was vertically released for mobilization. We dissected along the medial border of the platysma and sternocleidomastoid muscles, as well as the lateral border of the trachea and hypopharynx to expose the prevertebral space. We dissected the medial aspect of the bilateral longus coli muscles off the vertebral bodies, and inserted Koros self-retaining retractors. The teeth of the Koros retraction blade were placed just beneath the medial border of the longus coli to avoid injury to the hypopharynx or esophagus. We did not routinely use a longitudinal distraction system, such as a Caspar retractor. Therefore, there was no distraction across the intervertebral spaces. We used lateral intraoperative fluoroscopy for surgery-level localization, followed by a surgical microscope. We performed annulotomy, discectomy, and osteophytes removal using a high-speed hand drill, curettage, and Kerrison rongeurs. The posterior longitudinal ligament was resected. In the current study, we used a polyetheretherketone cage combined with an artificial bone graft for interbody fusion, which was completely covered by the National Health Insurance in Taiwan. No plates or screws were used for anterior fixation. The wounds were irrigated with saline and closed in a standard manner. We did not apply local steroids in the prevertebral space nor did we administer any intravenous steroids postoperatively. Typically, no drain is inserted after surgery. We administered postoperative antibiotics for 24 h, and the patient received appropriate analgesic medication, including acetaminophen and nonsteroidal anti-inflammatory drugs. Patients were discharged on the first or second postoperative day.

### Protocol

All patients had their swallowing function assessed using HRIM combined with the Dysphagia Short Questionnaire (DSQ; score: 0 to 18; low score, mild symptoms) at the three time points (PreOP, POD1, and POD7) (11). After the HRIM catheter was inserted through the pharyngeal-esophageal segment, we administered 5 mL of normal saline boluses on command via a syringe at >20 s minimum intervals. Every patient underwent the swallowing test ten times.

### Measuring Pressure-Flow Analysis Data From High Resolution Impedance Manometry

**Figure 3:**
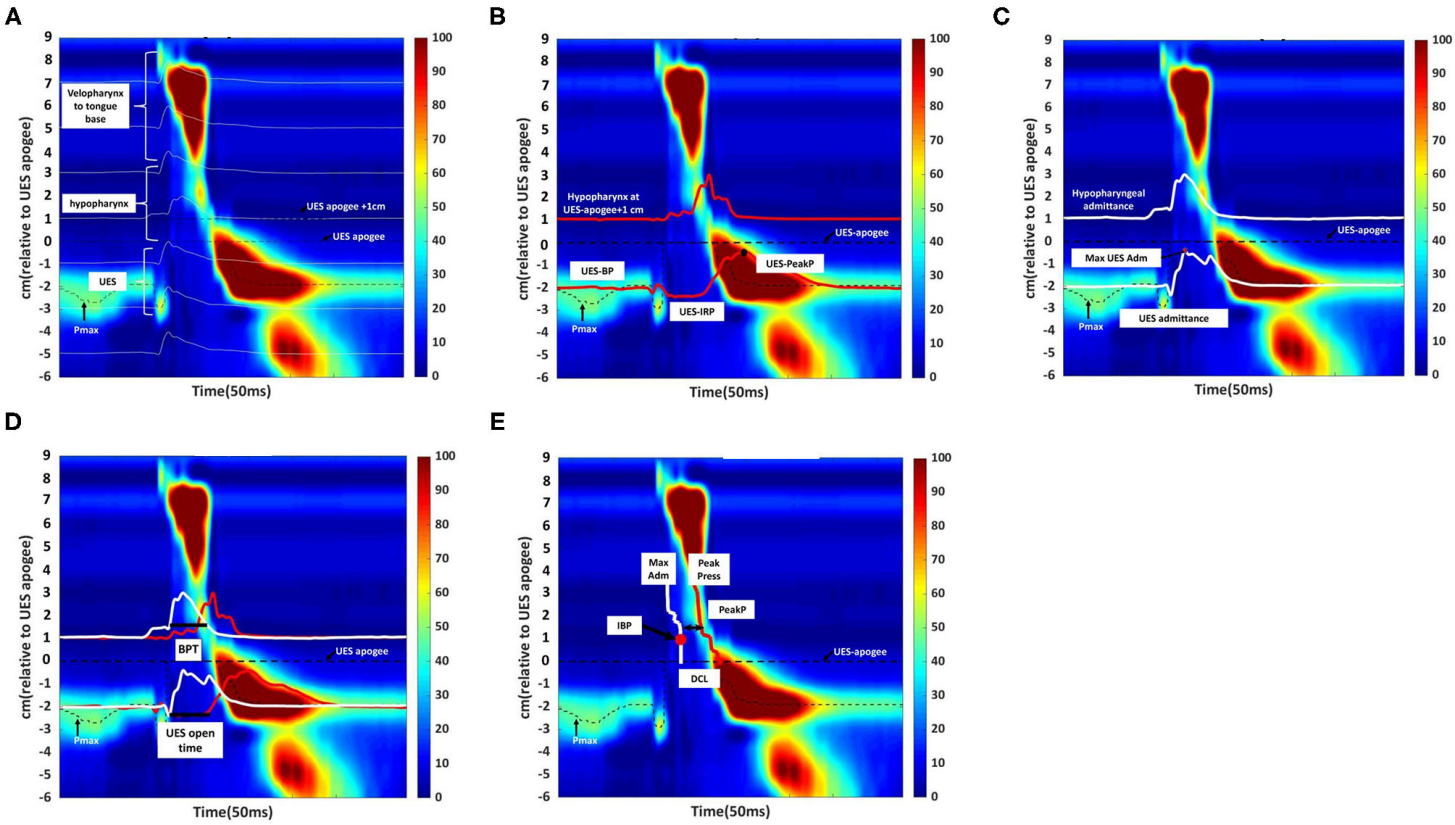
Swallowing function assessment via High -Resolution Impedance Manometry (HRIM). **(A)** Illustrative example of our data from the pressure sensors on the HRIM catheter (y-axis) over time (x-axis). The graph shows the oropharyngeal pressure topography, from the velopharynx to the tongue base, hypopharynx, and into the upper esophageal sphincter (UES). The high-pressure zone is the UES. The impedance channels from the velopharynx into the esophagus are indicated by white horizontal lines. Effective oropharyngeal muscle contraction relies on bolus transmission, during which the impedance level decreases. **(B)** Illustrative example of measurement of the parameters of hypopharyngeal and upper esophageal sphincter (UES) pressures. The upper red line represents the pressure waveform recorded at the hypopharynx during the swallow (apogee + 1 cm). The lower red line represents the UES pressure waveform, constructed from pressures recorded at the maximum UES pressure position over time. The mean pre-deglutitive UES basal pressure (UES-BP), UES integrated relaxation pressure (UES-IRP), and post-deglutitive UES peak pressure (UES-PeakP) can be identified at the lower red lines. **(C)** Illustrative example of measurement of the parameters of hypopharyngeal and upper esophageal sphincter admittance. Admittance (S) is the inverse product of impedance (Ω), i.e., S = 1/Ω. Admittance increases with bolus distension of the hypopharynx and UES. The maximum admittance within the UES (Max UES Adm) is represented by the maximum cross-sectional area of the lumen. The lower white line represents the UES admittance waveform, which is constructed from impedance recorded at the Pmax position over the swallowing period. The upper white line represents the admittance waveform at the hypopharyngeal position during the swallow (apogee +1 cm). **(D)** Illustrative example of measurement of bolus presence time (BPT) and upper esophageal sphincter (UES) open time. The lower section of the graph represents the UES region. The red line represents the UES pressure waveform, which is constructed from pressures recorded at the Pmax position over time. The white line represents the admittance waveform. UES open time was calculated from UES admittance and the pressure waveform, which were used together to define the onset of UES opening (based on the admittance upstroke in the UES), and UES closure (based on the pressure upstroke in the UES). The upper section of the graph represents the hypopharynx region (apogee + 1 cm). The red line represents the hypopharyngeal pressure waveform, and the white line represents the admittance waveform. The UES admittance level at the time of closure was used as the threshold to commence the hypopharyngeal admittance recording. The period when hypopharyngeal admittance exceeded this threshold was defined as the bolus presence time (BPT). **(E)** Illustrative example of measurement of the hypopharyngeal parameters. The white line indicates the maximum admittance (Max Adm) in the hypopharynx region. The red line indicates the mean value of the hypopharyngeal peak pressure (Mean Peak) in the hypopharynx region. Distension-contraction latency (DCL) reflects the time of maximum bolus distension and maximum contraction of the hypopharynx during the swallow. Hypopharyngeal intrabolus distension pressure (IBP) is defined as the pressure at maximum distension (at the position of Max Adm), 1 cm proximal to the UES apogee. The upper esophageal sphincter (UES) apogee is defined by visualization of the orad movement of the UES high-pressure zone to determine the highest position of the proximal edge of the high-pressure zone during swallowing. The Pmax position is defined as the position at the maximum pressure of UES.

HRIM data were calculated as the average of the ten swallowing tests completed by each patient. Figure 3A showed a high-resolution color pressure topography plot.

The acquisition system allowed the export of raw pressure and impedance data to a spreadsheet template (Microsoft Excel, Microsoft Corporation, Redmond, WA, USA). The data for each patient were analyzed using MATLAB 2019b (MathWorks Inc., Natick, MA, USA). All the parameters and their definitions are described in detail in Table 1 (11, 16). We used the following landmarks: (1) velopharynx and tongue base, (2) hypopharynx, and (3) UES apogee (14, 17, 18) (Figure 3A).

**Table 1:** Normative values for novel swallowing variables.

**Swallowing metrics**	**Meaning**	**Meaning when abnormal**	**Normal ranges (5 mL)**
Swallowing risk index (SRI)	Global swallowing function	Global swallowing dysfunction (>15)	0–11
Hypopharyngeal intrabolus pressure at 1 cm above UES (IBP, mmHg)	Measuring the hypopharyngeal pressure at the timing of the maximum size of hypopharyngeal opening achieved in the location of 1 cm above the UES	High resistance at the location of 1 cm above UES to transmit the bolus difficultly	−1–22
Hypopharyngeal mean peak pressure (PeakP, mmHg)	Hypopharyngeal contractility	Abnormal hypopharyngeal contractility	69–280
Hypopharyngeal distention contraction latency (DCL, ms)	The duration from the maximum opening size of hypopharyngeal muscle to peak contraction during bolus transmitting though the hypopharyngeal region.	discoordination between the hypopharyngeal muscle opening and contractility	317–598
Hypopharyngeal bolus presence time (BPT, s)	Duration of bolus presenting at hypopharyngeal region.	Prolonged duration due to ineffective hypopharyngeal muscle group contraction	0.50–0.98
UES maximum admittance (Max Adm, mS)	The size of UES opening at the time of the bolus through the UES smooth	Reduced size of UES opening at the time of bolus transmission through the UES difficultly	4.4–9.1
UES opening time	Effective UES contraction smoothly leading the bolus through the UES smoothly	Early or late bolus arriving because of the ineffective UES contraction	0.6–1.0
UES basal pressure (basal P, mmHg)	Pre-deglutitive tone	Reduced pre-deglutitive tone causing food into the oropharynx increasing probability of entering to the unprotected laryngeal opening	29–145
UES postdeglutitive peak pressure (PeakP, mmHg)	UES contractility	Reduced UES contractility	149–548
UES 0.25 integrated relaxation pressure (IRP, mmHg)	Enabling UES relaxation	UES opening restriction	−4–15
Velopharynx to tongue base contractile (VTI, mmHg.s.cm)	Velopharynx to tongue base contractility	Reduced velopharynx to tongue base contraction	300–700

*UES, upper esophageal sphincter; catheter using MMS, “solar GI” system*.

*mS, S was calculated to be 1/Ω. mS = S × 1,000; ms, millisecond*.

The pressure integral from the velopharynx to the tongue base (VTI) was derived by multiplying the mean pressure from the velopharynx to the tongue base (20 mmHg or higher) by the length of the velopharynx to the tongue base and then by the duration of the contraction in seconds (5). We defined the length of the velopharynx to the tongue base as the region between the superior margin of velopharyngeal contraction and the superior border of the hypopharynx (5). We calculated UES basal, peak and 0.25 s integrated relaxation pressure (IRP) (Figure 3B) (19, 20).

The UES and hypopharyngeal admittance is the inverse product of impedance (impedance: Ω, S = 1/Ω). The admittance rises with bolus distension of the hypopharynx and UES and the maximum admittance within the UES (Figure 3C). UES maximum admittance (UES Max Adm) is indicative of maximum cross-sectional area of the lumen (11).

The UES open time and hypopharyngeal bolus presence time (BPT) are presented in Figure 3D (11, 19). We assessed hypopharynx contractility using hypopharyngeal mean peak pressure (PeakP) (Figure 3E) (11). The hypopharyngeal intra-bolus distension pressure (IBP) was defined as the pressure recorded at maximum distension (1 cm proximal to the UES apogee, Figure 3E) (11). We defined the timing of flow to contraction as the average duration from hypopharyngeal muscle opening to contraction along the hypopharyngeal region [distension-contraction latency (DCL); Figure 3E] (11, 19). We defined the swallow risk index using the following formula (21):

SRI=(IBP × BPT)/(DCL + 1) × PeakP) × 100

### Statistical Analyses

We presented continuous data as means and standard deviations. Repeated measures ANOVA and Bonferroni *post-hoc* analyses were used to analyze the differences among the three time points. A *p*-value of *P* < 0.05 was considered statistically significant. Analyses were performed using SPSS (SPSS Inc., Chicago, IL, United States).

## Results

### Demographic Data

A total of 21 patients were eligible for inclusion in this study. We excluded four patients who declined to participate and three patients who refused to continue the HRIM swallowing test following the preoperative examination. Fourteen patients completed the study. Their detailed demographics information is presented in Table 2. The mean age was 59.84 ± 11.19 years and nine patients were men (64%). DSQ scores were 1.36 ± 1.22, 3.43 ± 1.95 and 2.36 ± 1.87 at PreOP, POD1, and POD7 time points (*P* < 0.01). DSQ scores at POD1 were significantly higher than PreOP or POD7. A total of 420 swallows were analyzed from these fourteen patients at all three time points.

**Table 2:** Demographics and contributing factors of 14 patients associated with perioperative swallowing changes.

**Case (Patient number)**	**Age, years/sex**	**Comorbidity**	**Level(s)**	**DSQ (PreOP/POD1/POD7)**	**Surgical duration (min)**
1	71/M	DM, hypertension	C3,4,5,6	2/0/1	147
2	42/M	nil	C4,5,6,7	0/4/1	251
3	74/F	nil	C4,5,6	0/4/0	146
4	61/M	hypertension	C5,6,7	2/2/0	121
5	64/M	renal cell carcinoma under target therapy	C6,7	4/3/4	197
6	58/M	chronic hepatitis B	C5,6	0/4/0	142
7	62/M	hypertension	C5,6	2/2/3	94
8	70/M	bladder cancer	C3,4,5,6,7	2/5/5	208
9	54/M	nil	C3,4	0/5/3	102
10	48/M	nil	C4,5,6,7	2/5/5	167
11	50/F	nil	C5,6	1/0/1	118
12	49/F	nil	C3,4	2/7/4	114
13	47/F	hyperlipidemia	C5,6	0/4/4	188
14	77/F	hypertension	C3,4,5,6	2/3/2	76

*M, male; F, female; PreOP, preoperative time point; POD1, postoperative day one; POD7, postoperative day seven; DM, diabetes mellitus*.

*C3, third cervical spine vertebrae; C4, fourth cervical spine vertebrae; C5, fifth cervical spine vertebrae; C6, sixth cervical spine vertebrae; C7, seventh cervical spine vertebrae; DSQ, dysphagia short questionnaire*.

### Pressure and Impedance Changes Measured at D0, D1 and D7 by HRIM

**Figure 4:**
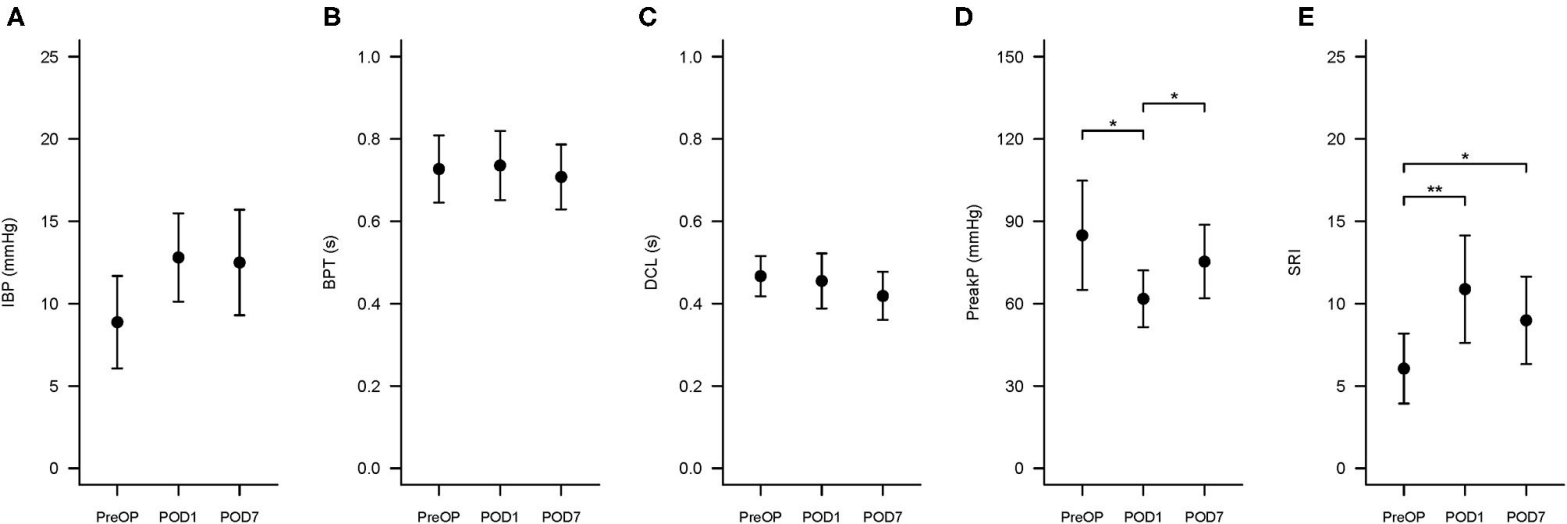
Swallowing Risk Index (SRI) and associated hypopharyngeal parameters changes at three time points. The three time points are PreOP (preoperative day), POD1 (postoperative day 1), and POD7 (postoperative day 7). The formula to calculate SRI is (IBP × BPT)/(DCL × PeakP) ×100. **(A–E)** Presented the error bar of IBP, BPT, DCL, PeakP, and SRI at PreOP, POD1 and POD7, respectively. *P < 0.05; **P < 0.01. SRI, Swallowing risk index; IBP, intrabolus pressure (mmHg); BPT, bolus present time (s); DCL, hypopharyngeal distention contraction latency (s); PeakP: hypopharyngeal mean peak pressure (mmHg).

The changes in swallowing variables at the three time points are presented in Table 3 and Figure 4. The swallowing risk index (SRI) was significantly higher on POD1 (10.88) than on PreOP (6.06) and POD7 (8.99). A relative high SRI at POD1 (16.05) and POD7 (15.09) in patient number 9 (Table 2) with a higher preoperative SRI (12.86>11) was found. This patient had a low preoperative value of PeakP (61.88 mmHg). This indicates that the weak hypopharyngeal muscle groups are insufficient to transmit the bolus into the UES so that the bolus accumulates in the hypopharyngeal region and increases aspiration risk. The mean values of the SRI and other associated parameters for all patients are presented in Figure 4.

**Table 3:** Pharyngeal and upper esophageal sphincter parameters among HRIM swallow tests while in a neutral sitting position.

**Metric**	**PreOP (*n* = 14)**	**POD1 (*n* = 14)**	**POD7 (*n* = 14)**	***P***
Swallow Risk Index	6.06 (3.71)	10.88 (5.69)^*^	8.99 (4.64)^‡^	0.023
Hypopharyngeal Mean Peak Pressure (mmHg)	84.9 (34.7)	61.8 (18.0)^*^	75.3 (23.4)^†^	0.045
Velopharynx to tongue base contractile integral (mmHg.s.cm)	553.3 (284.8)	464.9 (139.1)	474.2(206.9)	0.14
UES basal pressure (mmHg)	19.8 (15.4)	21.3 (8.1)	21.6 (16.1)	0.80
UES peak Pressure (mmHg)	112.9 (49.3)	80.4(30.0)^*^	105.6(59.1)^†^	0.017
UES open time (s)	0.89 (0.30)	0.93(0.30)	0.84 (0.22)	0.31
UES maximum admittance (mS)	3.85 (0.80)	3.53 (0.64)	3.99 (0.67)	0.07
UES 0.25 s integrated relaxation pressure (mmHg)	19.4 (9.2)	25.7 (13.0)	22.8 (8.2)	0.21
The average latency from hypopharyngeal maximum distension to peak contraction (DCL, ms)	466.91 (85.20)	455.10(116.77)	418.65(101.65)	0.13
Hypopharyngeal intra-bolus pressure at 1 cm above UES (mmHg)	8.9 (4.9)	12.8 (4.7)	12.5 (5.6)	0.05
Bolus present time (s)	0.73 (0.14)	0.74 (0.15)	0.71 (0.14)	0.52

*Data are presented as mean (standard deviation). HRIM, high-resolution impedance manometry; UES, upper esophageal sphincter; PreOP, preoperative time point; POD1, postoperative day one; POD7: postoperative day seven; n, number of wet swallows for evaluation*.

*mS, S was calculated to be 1/Ω. mS = S × 1000; ms: millisecond*.

* *p < 0.05, POD1 compared to PreOP*;

† *p < 0.05, POD7 compared to POD1*;

‡ *p < 0.05, POD7 compared to PreOP*.

For hypopharyngeal parameters, the PeakP indicating hypopharyngeal contractility was significantly lower at POD1 (61.8 mmHg) than PreOP (84.9 mmHg) and POD7 (75.3 mmHg; Tables 1, 3). UES peak pressure expressing UES contractility on POD1 (80.4 mmHg) was significantly lower than PreOP (112.9 mmHg) and POD7 (105.6 mmHg). Bolus presence time (BPT) and average latency from the hypopharyngeal maximum distension to peak contraction (DCL), implying the ability of bolus flow through the hypopharyngeal region smoothly presented no significant difference among the three time points (Tables 1, 3). Velopharynx to tongue base contractility presenting the muscle power at this region showed no significant differences among the 3 days (Table 3).

For evaluating UES effective opening (Tables 1, 3), UES maximum admittance (Max Adm) and UES open time showed no significantly changes at POD1 and POD7 compared to the data at PreOP (Tables 1, 3). UES 0.25 integrated relaxation pressure (IRP) indicating the capability of UES relaxation presented no significantly changes among these three time points (Tables 1, 3). UES basal pressure (basal P) indicating pre-deglutitive muscle tone showed no significantly difference among these time points (Tables 1, 3).

## Discussion

In this study, we were able to use HRIM to assess the perioperative swallowing functions in ACSS patients and detect changes in swallowing. Multiple HRIM impedance channels can be used to measure a liquid-based bolus that generates a low impedance signal, consistent with pharyngeal peristalsis (22). Our results showed that in ACSS patients without perioperative swallowing problems, the major factor interfering with swallowing was the effective contractility of the UES and hypopharyngeal muscle groups. When this contractility is less effective and associated with increasing SRI, these symptoms may be associated with surgical traction and resolve within seven days. HRIM measurements may play a particularly important role in differentiating the mechanisms of postoperative swallowing dysfunction and assessing preoperative swallowing function as a reference for patients with a high risk of developing postoperative dysphagia.

Our results showed that the measurements from the HRIM, including the SRI, provided more detailed information about changes in swallowing than the DSQ. A change in the DSQ score from 1.39 to 1.95 represented a slight change, without further information on swallowing, clinical impact or warning. However, SRI is a combined index that includes IBP, BPT, DCL and PeakP. These measurements indicate whether the hypopharyngeal peristalsis can transmit the bolus smoothly from the oropharyngeal region through the hypopharyngeal region into the esophagus. Because bolus retention in the hypopharyngeal region increases the possibility of the bolus entering the unprotected airway, there is an increased SRI and risk of aspiration. Clinicians should be aware that patients have a risk of aspiration risk when their SRI exceeds the normal range (>11) (11). One of our patients presented with a high SRI and increased data on PODs 1 and 7. We suspect that this patient had a relatively high preoperative intrabolus pressure, indicating a relatively high resistance to pharyngeal outflow. This would explain the high SRI after surgery. In previous studies, patients with high preoperative aspiration risk were assumed to have a high postoperative aspiration risk well (23). This indicates that these patients may have had swallowing dysfunction, but that the causative mechanisms were not identified preoperatively. This was because the treating physicians had not used an objective method for identifying swallowing dysfunction mechanisms. Early detection and assessment of swallowing dysfunction, and treating the root causes, may reduce complications, such as aspiration and its sequelae (10). In our study, using HRIM helped us to identify the underlying mechanisms. Further investigations and management, including postoperative swallowing education, should also be performed.

Generally, rapidly changing and widely varying pressures across the pharyngoesophageal segment make it difficult to conduct traditional manometry, which uses only a few transducers (24). In the pull-through techniques used in these traditional methods, sensors may be displaced from the UES high-pressure zone after breathing or coughing (25). With HRIM, multiple sensors and impedance channels allow the measurements to be analyzed in an integrated fashion (26). This approach has a high intra- and inter-rater reproducibility (9, 14). Furthermore, this catheter-based approach is clinically reliable and objective method for assessing swallowing function (9).

Historically, videofluoroscopy has been the most widely used technique for evaluating swallowing anatomy and physiology (27). However, besides the radiation exposure from associated with this technique (9), it cannot measure the contractility of the subcomponents (the velopharynx, tongue base and hypopharynx) (17). If these regions are combined, the composite measurement may appear “normal,” even in the presence of focal velopharynx-to-tongue base or hypopharyngeal muscle weakness. For example, increased viscosity of the barium preparation or higher bolus volume can lengthen bolus transmission time through the pharynx (28, 29). It is important to simultaneously evaluate muscle power and the condition of fluid bolus transmission. Furthermore, inter-rater reliability can vary widely depending on the analysis method for radiological images and the consistency of the bolus being swallowed (9).

The swallowing parameters measured via HRIM in our study (DCL, UES open time and UES maximum admittance in particular) showed fluid boluses passing smoothly and directly through the esophagus in all of our patients. These data were similar to patients without objective oropharyngeal dysphagia (11). These parameters simultaneously took pharyngeal pressure and bolus transmission into account, meaning that HRIM provided more detailed measurements than videofluoroscopy (11). Despite observing hypopharyngeal and UES muscle weakness and increasing SRI at POD1 (although these were within normal limits), the other parameters remained unaffected. This indicates that the weak UES and hypopharyngeal muscles were still sufficient to propel the bolus through the pharynx and UES at POD1 and thus not cause the bolus to accumulate around these regions and increase aspiration risk. We found that our measurements of UES and pharyngeal function were similar to those in previous studies of healthy volunteers (11). In addition, our data on UES pressure changes including UES opening from HRIM was similar to those of Nelson et al. (30). Our hypopharyngeal pressure data, however, were different from those of Rosen et al. (31). Rosen et al. used a different HRIM catheter and swallowing volume, resulting in the different pressure values they obtained in the hypopharyngeal region (11). Our catheter had 36 circumferential sensors, while the one used by Rosen et al was three-dimensional could measure four directional pressure changes, along with the mean pressures. In our study, patients swallowed 5 mL in every swallow test, but the volume was 10 mL in the study by Rosen et al.

The DSQ scores we recorded increased from the PreOP measurement (mean: 1.36) compared with POD1 measurement (mean: 3.43) and then decreased again by POD7 (mean: 2.36). The potential range of DSQ scores is from 0 to 18. The lower scores represent the milder symptoms (32). Overall, the mean DSQ scores we recorded were low. The small changes observed may indicate some swallowing discomfort, but not dysphagia or any swallowing problems. The HRIM measurements produced normal values, suggesting that none of the patients had any objective swallowing problems.

Our study has several limitations. First, because of the close arrangement of the pharyngeal structures, the 1 cm HRIM intervals used can detect detailed contractions in adjacent anatomical structures (33). However, it is difficult to differentiate between the velopharynx and mesopharynx (which includes the tongue base) as in the previous study (31), because deglutitive waves sometimes occur simultaneously (33). Therefore, the swallowing specialist assumed that the contractions were simultaneous (34). Second, we used only one bolus condition. Our results indicated a pattern of increasing IBP and reduced admittance. This phenomenon may be affected by the volume and viscosity of the bolus (35), however, but this could not be confirmed in this study. We are planning further research to address this question. Third, the ACSS was not performed at the same point on cervical spine in all patients, Performing the surgery at different points on the spine affects different locations on the pharynx, and it is difficult to elucidate the different effects this would have. However, regardless of the point on the cervical spine at which the surgery was performed, all of our patients received traction injuries during their ACSS (7, 36). We were able to use the HRIM to detect the changes perioperatively. We plan to investigate further effect of the different locations of surgery. Fourth, our patients presented slight perioperative changes in swallowing. Previous studies have identified factors that increase the risk of patients exhibiting swallowing disorders after cervical spine surgery, such as prolonged intubation or female gender (4, 37). We plan to investigate patients with actual swallowing disorder in the future. Fifth, the HRIM results were analyzed retrospectively. However, this did not affect our results because our analysis was based on published and validated methods (11). Sixth, the small sample size may have affected the results. However, the HRIM findings and patterns we observed n our patients were homogenous. Seventh, HRIM insertion is invasive, painful, and resulted in our sample size (only 14 patients agree to participate in these investigations). This also was reflected in the high withdrawal rate (~25%).

## Conclusion

With similar trends in DSQ and SRI, swallowing functions were significantly decreased on POD1 because of decreasing hypopharyngeal and UES contractility but recovered toward preoperative state on POD7 after ACSS. Applying HRIM measurement is superior to DSQ on detecting possible mechanisms and monitoring recovery from global swallowing dysfunction, and also a feasible method for objectively assessing swallowing function perioperatively.

## Data Availability Statement

The raw data supporting the conclusions of this article will be made available by the authors, without undue reservation.

## Ethics Statement

The studies involving human participants were reviewed and approved by 201901089RINC the Ethics Committee of the National Taiwan University Hospital. The patients/participants provided their written informed consent to participate in this study.

## Author Contributions

C-JL and F-YT contributed to the study concept, design, and data acquisition. C-JL contributed to the data analysis and interpretation, drafting of the manuscript, and statistical analysis. Y-JC and C-YW revised the manuscript. All the authors approved the final version of the manuscript. All authors contributed to the article and approved the submitted version.

## Funding

This research was partially supported by National Taiwan University Hospital Grant (110-N4955 and 110-N4610).

## Conflict of Interest

The authors declare that the research was conducted in the absence of any commercial or financial relationships that could be construed as a potential conflict of interest.

## Publisher's Note

All claims expressed in this article are solely those of the authors and do not necessarily represent those of their affiliated organizations, or those of the publisher, the editors and the reviewers. Any product that may be evaluated in this article, or claim that may be made by its manufacturer, is not guaranteed or endorsed by the publisher.

## Abbreviations

ACSS: Anterior cervical spine surgery
DCL: Hypopharyngeal distension-contraction latency
HRIM: High resolution impedance manometry
IBP: Intrabolus distension pressure
PeakP: Hypopharyngeal mean peak pressure
POD1: Postoperative day 1
POD7: Postoperative day 7
PreOP: Preoperative time point
SRI: Swallowing risk index
UES: Upper esophageal sphincter
VTI: Velopharyx-to-tongue base contractile.
